# Meridional Amblyopia and Spectacle Correction: A Prospective Interventional Study of Children Aged 4–11 Years

**DOI:** 10.7759/cureus.67549

**Published:** 2024-08-22

**Authors:** Mutahir Shah, Satheesh Babu Natarajan, Nafees Ahmad

**Affiliations:** 1 Ophthalmology, Lincoln University College, Kuala Lumpur, MYS; 2 Health Sciences, Lincoln University College, Kuala Lumpur, MYS; 3 Genetics, Institute of Biomedical and Genetic Engineering, Islamabad, PAK

**Keywords:** astigmatism treatment, with the rule astigmatism, spectacle correction, pediatric refraction, meridional amblyopia

## Abstract

Background

Amblyopia, often linked to high astigmatism in children, presents challenges in understanding the contributing factors and visual outcomes.

Methods

A total of 132 samples were included in this prospective pre- and post-interventional study using the purposive sampling technique. Descriptive statistics were applied for age, gender, uncorrected visual acuity, corrected visual acuity, type, and magnitude of astigmatism. A repeated measure ANOVA was used, and a paired t-test was also done for groups with corrected visual acuity in meridional amblyopia at two follow-ups spaced six months apart. Logistic regression was used to identify the association between astigmatism types and patients’ recovery from amblyopia after intervention.

Results

The age of participants was 4-11 years with meridional amblyopia. Initial findings showed a mean uncorrected visual acuity of 0.73 LogMAR in the right eye (RE) and 0.71 LogMAR in the left eye (LE), improving significantly to 0.35 LogMAR post-intervention in both eyes. The calculated mean difference between the first correction and the first follow-up was 0.12 and 0.13 LogMAR, while it was 0.20 and 0.21 LogMAR in the RE and LE, respectively, at the second follow-up. Spherical refractive errors averaged +0.93 DS, with mean cylindrical refraction indicating predominant with-the-rule corneal astigmatism (-3.46 DS). We observed a significant improvement in visual acuity (p-value < 0.001) and an increase in the magnitude of cylindrical prescription (p-value < 0.001). However, astigmatism types are not associated with response to therapy.

Conclusion

The study concluded that early detection of meridional amblyopia and early intervention with spectacles significantly improve visual acuity.

## Introduction

Amblyopia, commonly known as "lazy eye," is a visual development disorder in which the eye fails to achieve normal visual acuity, even with the use of prescribed spectacles or contact lenses [1]. A decrease in best-corrected visual acuity (BCVA), a measurement of the two-line difference in visual acuity, defines amblyopia operationally [2]. The prevalence of amblyopia varies in different geographical regions and age groups. The prevalence of amblyopia in children is between 2.6% and 7% worldwide, with a pooled prevalence of 4.3% [3].

Amblyopia is classified into three major types: refractive, strabismic, and stimulus deprivation. Refractive amblyopia is a major type of amblyopia, which is classified into hypermetropic, myopic, and astigmatic amblyopia. Astigmatic amblyopia, also known as meridional amblyopia (MA), is a type of refractive amblyopia that occurs in astigmatism.

Astigmatism is an optical condition in which parallel rays of light coming from infinity are refracted differently by the two curvatures of the cornea or lens. Astigmatic refractive status can lead to several visual disturbances. These include the clarity of grating patterns, the ability to recognize fine details, contrast sensitivity, the sense of alignment judgment, and stereopsis, and these may even lead to the development of amblyopia [4,5]. Astigmatism may be isoametropic if the refractive error between the two eyes is the same or anisometropic if it is different in both eyes. Astigmatism may lead to amblyopia if the refractive error is >2.50 DC in isoametropic conditions, while anisometropic condition requires >1.50 DC [6].

This difference in visual acuity results in blurred images, causing confusion in the visual cortex. The brain perceives confusion when there is a disparity among the images seen by each eye, causing it to suppress the more blurred image. This suppression can lead to the development of amblyopia. The condition not only affects visual acuity but also has potential implications for the child's developmental, educational, and social outcomes. The critical period of visual development is around eight years in children [7]. Early detection of MA and early correction are pivotal to improving visual outcomes and preventing the progression of visual impairment. Spectacles are still a non-invasive intervention and have been widely used for correction, yet the evidence on their efficacy specifically for MA in children is both sparse and fragmented.

The goal of this prospective interventional study is to address the gaps in our knowledge by examining how effectively corrective glasses improve visual acuity in children aged 4-11 who have been diagnosed with MA. The study focuses on the opportunity for effective intervention that will align with the neuroplasticity of the developing brain, within this critical developmental period. The study will not only contribute to the scientific understanding of MA correction but also provide guidelines for clinicians to improve the quality of vision, educational performance, and social integration for affected children.

## Materials and methods

A prospective pre- and post-interventional study was conducted in the department of ophthalmology at a tertiary care hospital in Islamabad, Pakistan. The study was approved by the hospital ethical committee under letter no: AMC-HI-PUB-ERC/March 23/24. Informed written consent was obtained from the participants/guardians of minors and children aged 7-11 years. All steps were followed as per the guidelines of the Declaration of Helsinki. The sampling technique was nonprobability-purposive sampling. The aim of adopting this technique was to address the specific condition of MA and its response to optical treatment. The study lasted about 12 months, from January 15, 2023, to January 15, 2024. The following formula was used for estimating sample size in proportions:

$n\ =\ Z^2 \times p \times \frac{(1-p)}{d^2}$

where n is the required sample size, Z is the Z-score corresponding to the desired confidence level, p is the estimated prevalence of the condition, and d is the margin of error.

For a 95% confidence level, the Z-score was approximately 1.96. The prevalence (p) was selected from a previously published study in Pakistan for MA, which was 9.5% or 0.095 [8]. We assumed a margin of error (d) of 5% or 0.05. The calculated sample size was 129. However, we included 140 individuals in the study to overcome the follow-up losses. All patients diagnosed with MA were included. Amblyopia was defined as the BCVA of less than 0.3 log MAR in one or both eyes [9]. The MA can be further classified based on visual acuity as mild if BCVA is 0.2-0.3 log MAR, moderate if BCVA is 0.3-0.8 log MAR, and severe if it is more than 0.8 log MAR [10]. Children aged 4-11 years were included. This age group was selected because the sensitive period of development of amblyopia varies between three and eight years, and responses to amblyopia treatment obtained good results during this age group [7]. A study showed that it could be effective in teenagers as well [11]. Those with systemic illness, opacities of the refracting mediums, ectasias, trauma, cataracts, and h/o surgery (refractive, cataract, pterygium, squint, and retinal detachment (RD)) were excluded.

All patients fulfilling the inclusion criteria were examined for visual acuity uncorrected and best corrected, using the Early Treatment Diabetic Retinopathy Study (ETDRS). Noncycloplegic autorefraction was performed using an autorefractometer (NIDEK ARK 1, Japan) and retinoscopy at 67 cm using a Streak Retinoscope (NIETZ, Tokyo, Japan). The patient's pupils were then dilated using 1% cyclopen eye drops that were instilled three times with a 5-10-minute gap. The retinoscopy was conducted after 45 minutes using the retinoscopy technique. Post-cycloplegic autorefraction was performed, and the results were documented. To avoid the effects of dilation, the patients were called for post-mydriatic/cycloplegic refraction after two days. Then refraction was done, and glasses were prescribed. K (keratometry) readings were measured using an automated keratometer and confirmed by corneal topography using an optical coherence tomography (OCT)-based OPTOPOL Revo NX-130 instrument (Kraków, Poland). Corrected visual acuity was documented along with K readings and the magnitude of refractive error. A detailed fundus examination was performed using a slit lamp biomicroscope. The patients were followed for 12 months, with a follow-up interval of six months, and the results were documented on a printed proforma.

Data analysis was conducted using SPSS version 21 (IBM Corp., Armonk, NY). Categorical data, such as gender and type of astigmatism, were presented in graphical form and as percentages. Descriptive statistics were calculated for uncorrected and corrected visual acuity, pre- and post-cycloplegic refraction (spherical and cylindrical), corneal astigmatism, residual astigmatism, etc. Repeated ANOVA was applied, as the single variable visual acuity was observed at four separate time points. A paired t-test was then applied in groups to find any statistical association at various time points and any relationship between cylindrical refractive error changes over time. The 0.05 confidence interval was used significantly for all the results. Logistic regression was used to assess the association between different types of astigmatism and the response to amblyopic therapy.

## Results

The study included 140 participants; however, some patients were dropped during the follow-up. The study was completed with 132 participants. The mean age of participants was 6.47 ± 1.7 years (range: 4-11 years). The study participants included both genders: 57.86% were males and 42.14% were females. The descriptive statistics showed a comprehensive presentation of 132 individuals, including demographic characteristics, pre- and post-visual acuity measures, and refractive errors (Table 1).

**Table 1:** Descriptive statistics showing mean and standard deviation for all variables (n = 132) This table presents the descriptive statistics for 132 patients, including mean, standard deviation, minimum, and maximum values for key variables. SC: Uncorrected visual acuity; CC: Corrected visual acuity; R: Right eye; L: Left eye.

Parameters	Mean ± SD		Range	
			Minimum	Maximum
Age	6.47 ± 1.7		4 years	11 years
Visual acuity R eye (LogMAR)	SC	0.73 ± 0.14	0.36	1.20
	CC	0.35 ± 0.05	0.24	0.56
Visual acuity L eye (LogMAR)	SC	0.71 ± 0.14	0.30	1.20
	CC	0.35 ± 0.05	0.26	0.54
Pre-cyclo sphere (DS)	RE	0.92 ± 1.75	5.75	-6.50
	LE	0.95 ± 1.62	5.75	-6.75
Post-cyclo sphere (DS)	RE	1.63 ± 1.99	6.25	-5.5
	LE	1.71 ± 1.72	6.25	-6.0
Pre-cyclo cylinder (DC)	RE	-3.44 ± 0.98	-2.0	-6.50
	LE	-3.48 ± 1.06	-2.0	-6.0
Post-cyclo cylinder	RE	-3.45 ± 0.99	-2.0	-6.50
	LE	-3.47 ± 1.03	-2.0	-6.0
Pre-cyclo spherical equivalent (DS)	RE	-0.79 ± 1.78	3.75	-8.25
	LE	-0.78 ± 1.71	3.75	-8.38
Post-cyclo spherical equivalent (DS)	RE	-0.09 ± 1.9	4.50	-6.50
	LE	-0.141 ± 2.0	5.25	-6.37
Corneal astigmatism RE (DC)	RE	-3.19 ± 0.91	-1.50	-6.0
	LE	-3.19 ± 0.87	-1.0	-5.75
Residual astigmatism (DC)	RE	-0.26 ± 0.49	1.0	-2.50
	LE	-0.32 ± 0.56	0.75	-2.50

The mean uncorrected visual acuity in the right eye (RE) was 0.73 ± 0.14 and 0.71 ± 0.14 Log MAR in the left eye (LE) (range: 0.36-1.20 and 0.30-1.20). The mean uncorrected visual acuity was slightly better in the LE than in the RE. The mean corrected visual acuity was 0.35 ± 0.05 SD in the RE and 0.35 ± 0.05 SD Log MAR in the LE (range: 0.24-0.56 and 0.26-0.54). The calculated mean difference for visual acuity was first corrected. While it was 0.12 in the RE and 0.13 in the LE at the first follow-up, it increased to 0.20 and 0.21 log MAR at the first and second follow-ups, respectively (Table 2).

**Table 2:** Repeated measure ANOVA applied to visual acuity status at different time points before and after the intervention This table presents the results of repeated measures ANOVA evaluating visual acuity in meridional amblyopia cases across different time intervals (pre-intervention, post-intervention, first follow-up, and second follow-up). The F-values for the right and left eyes were 1415.26 and 1660.17, respectively, with a p-value of <0.001, indicating statistically significant differences in visual acuity at different time points. RE: Right eye; LE: Left eye.

Source		Type III sum of squares	Mean square	F-value	Significance
Visual acuity RE	Greenhouse-Geisser	25.9	16.9	1415.26	<0.001
Visual acuity LE		25.46	16.61	1660.17	

The mean spherical refractive error was +0.92 ± 1.75 in the RE and 0.95 ± 1.62 DS in the LE. The mean pre-cyclo spherical equivalent was -0.79 ± 1.78 DS in the RE, ranging from -8.50 DS to 3.75 DS, while it was -0.78 ± 1.71 in the LE, ranging from -8.38 DS to 3.75 DS. The mean pre-cyclo cylindrical refraction for the RE was -3.44 ± 0.98 SD, while it was -3.48 ± 1.06 for the LE. The range was -2.0 to -6.50 in the RE and -2.0 to -6.0 in the LE, respectively. Residual astigmatism ranged from -2.50 to 1.00 DC. Astigmatism was mostly corneal, as was the rule in nature. The mean value of corneal astigmatism was -3.19 ± 0.91 in the RE and -3.19 ± 87 SD in the LE. This large dataset gives us useful information about the visual traits of the sample population. It also helps to learn more about the relationship between refractive errors and visual acuity in MA.

**Figure 1:**
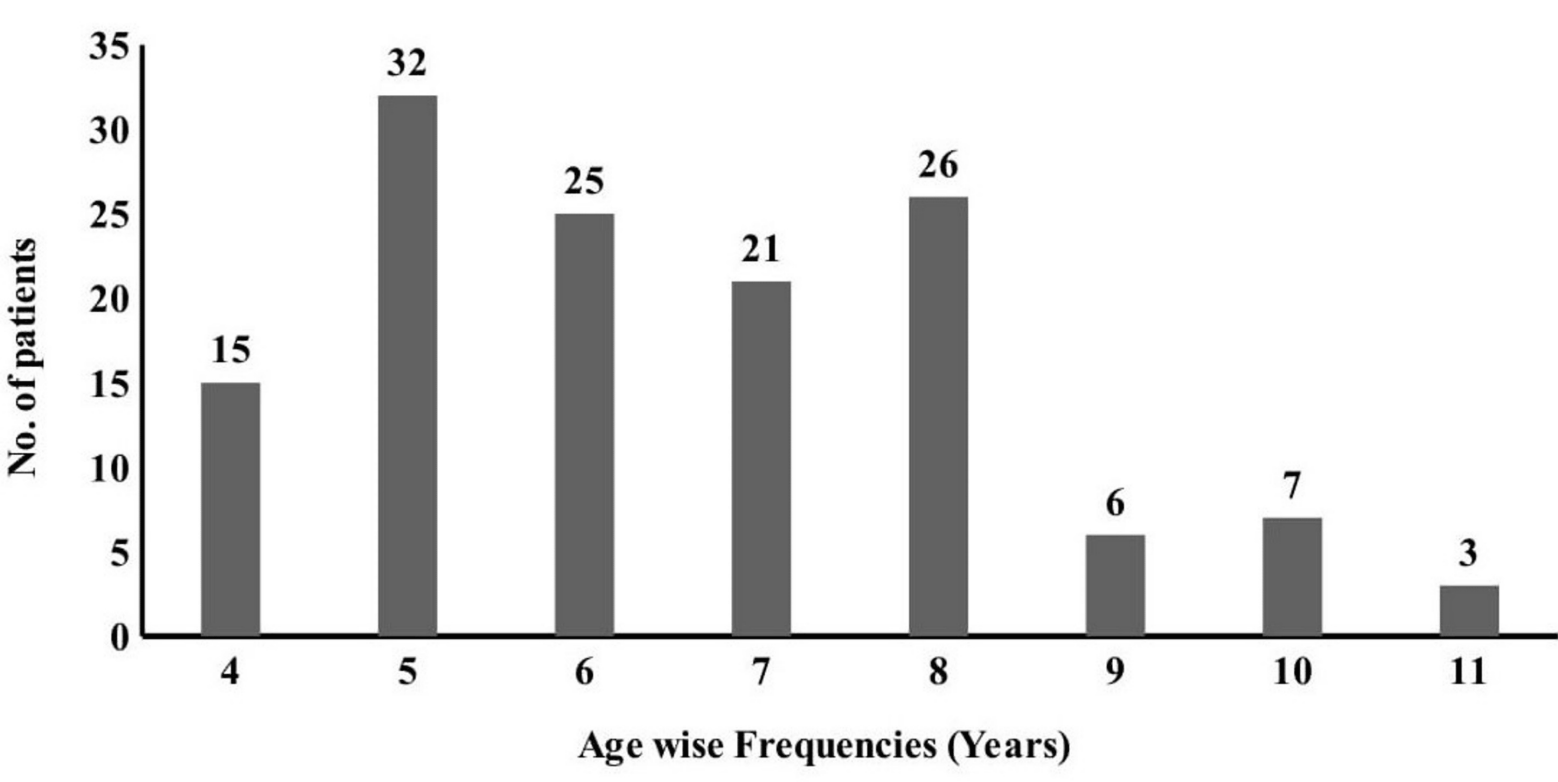
Age-wise representation of children with astigmatism The graph represents the frequency distribution of children diagnosed with meridional amblyopia, with the majority in the four- to eight-year age group. This age range is critical for oculo-neurological development.

Figure 1 presents the age-wise distribution of children with astigmatism and MA. Astigmatism is further classified as isoametropic, where the inter-eye difference is <1.50 DS, and anisoametropic, where the inter-eye difference is >1.50 DC.

**Figure 2:**
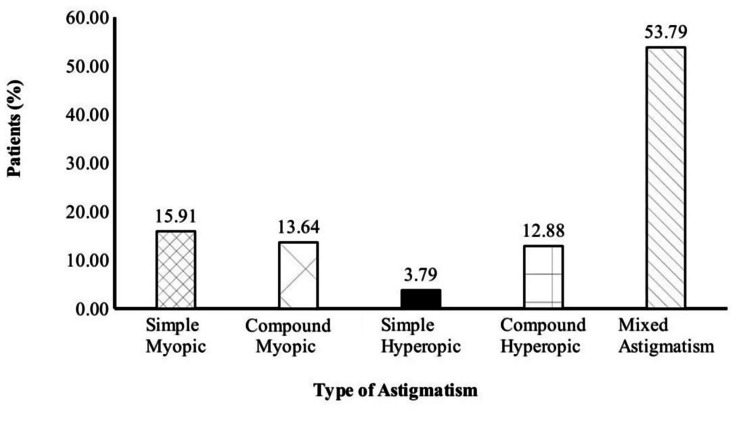
Graphical presentation of astigmatism types associated with meridional amblyopia This figure shows the percentage distribution of astigmatism types based on foci associated with meridional amblyopia. Mixed astigmatism is the most prevalent type, accounting for 53.79% of cases.

Based on loci, the type of astigmatism was further classified. Most cases of MA were related to the type of mixed astigmatism, followed by other types, with the least common being simple hyperopic astigmatism (HA) (Figure 2).

**Figure 3:**
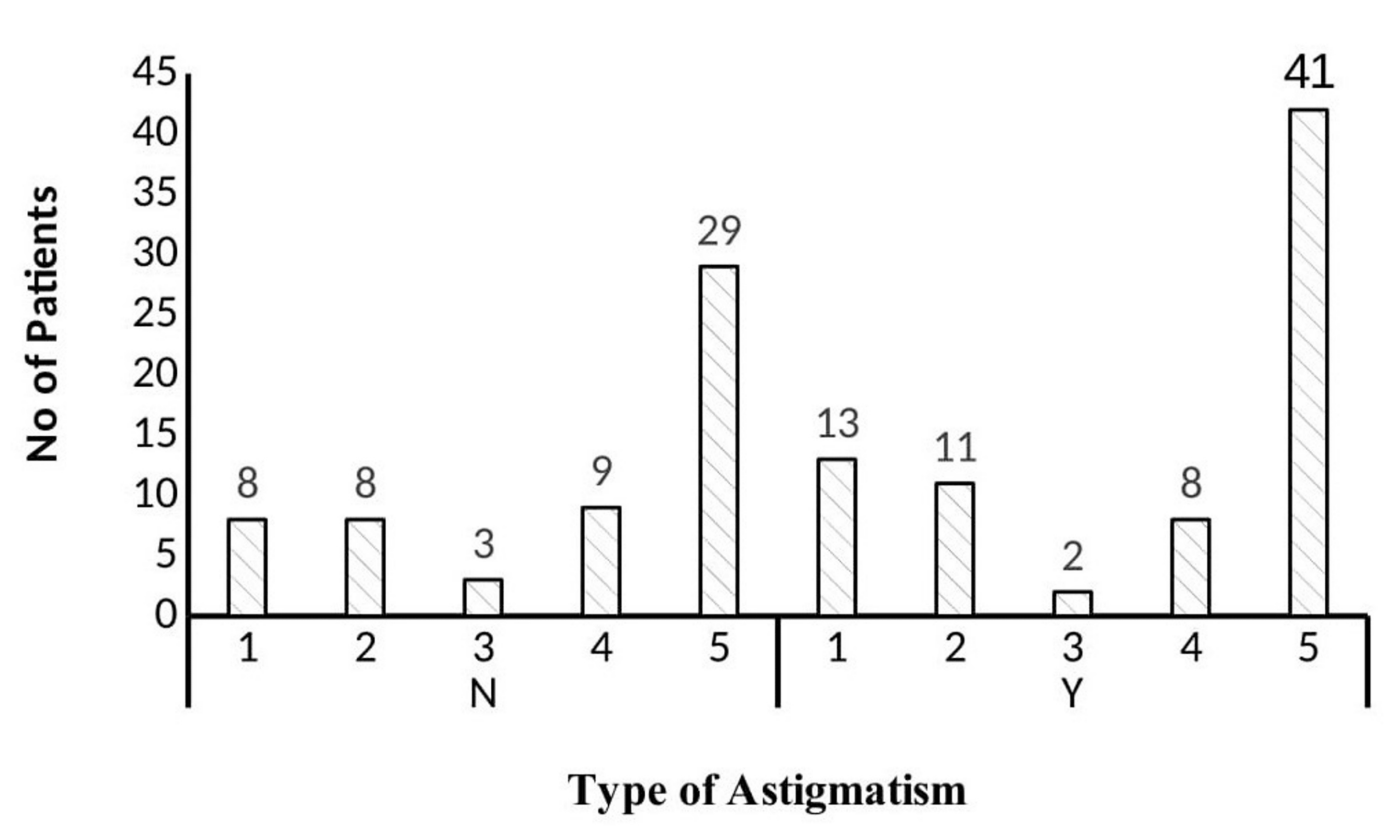
Children with and without normal vision in meridional amblyopia This figure presents the proportion of children with meridional amblyopia who achieved normal vision versus those who did not, after one year of spectacle correction.

Figure 3 shows the results of the spectacle intervention for amblyopia recovery, with both those who had achieved normal vision and those who still had some degree of amblyopia reflected (Figure 3).

The results of repeated measure ANOVA showed a statistically significant effect of the intervention on visual acuity, with a p-value of <0.001, indicating significance at a 95% confidence interval (CI) (Table 2). In other words, there is convincing evidence to suggest a significant difference in visual acuity across different conditions or time intervals. The paired t-test analysis also showed a significant difference among groups with a p-value of <0.001 and 0.05 CI (Table 3), indicating significant improvement in visual acuity after the intervention.

**Table 3:** Visual acuity recorded pre- and post-intervention (spectacles) on the two follow-ups (paired t-test statistics) The results of the paired t-test show mean differences in visual acuity (VA) for uncorrected VA, corrected VA, and corrected VA at both six- and 12-month follow-ups. The significance level was set at p < 0.05, with t-values indicating highly significant differences (p < 0.001) in visual acuity across the different measurement points. SC: Uncorrected visual acuity; CC: Corrected visual acuity; #: Six-month follow-up; $: 12-month follow-up.

Mean difference (n = 132)	Mean difference ± Standard deviation		95% confidence interval of the difference		t-value	Sig. (two-tailed)
			Lower	Upper		
Visual acuity SC vs. CC	RE	0.37 ± 0.13	0.35	0.39	33.00	<0.001
	LE	0.35 ± 0.12	0.33	0.37	33.40	
Visual acuity CC vs. first follow-up (180 days^#^)	RE	0.12 ± 0.07	0.11	0.13	20.32	
	LE	0.13 ± 0.06	0.12	0.14	26.00	
Visual acuity CC vs. second follow-up	RE	0.20 ± 0.07	0.18	0.21	30.0	
	LE	0.21 ± 0.06	0.20	0.22	36.55	
Visual acuity first follow-up vs. second follow-up (360 days^$^)	RE	0.071 ± 0.04	0.06	0.07	19.3	
	LE	0.078 ± 0.04	0.07	0.08	20.4	

A significant difference was observed in the values of cylindrical prescriptions over a one-year period, which showed that the magnitude of the cylinder was increasing significantly (Table 4). The odds among the type of astigmatism and visual outcome with spectacles were calculated, but the results were insignificant (Table 5). It was important to note that none of the astigmatism types showed a statistically significant difference in the odds of amblyopia response to intervention, as indicated by p-value > 0.05. The results revealed that there was no association between response to amblyopia after intervention and the types of astigmatism.

**Table 4:** The results of post-cyclo cylindrical prescription change over a period of six- and 12-month follow-ups (n = 132) This table shows the changes in post-cycloplegic cylindrical prescription at six- and 12-month follow-ups. The significance level was set at p < 0.05, with t-values indicating highly significant (p < 0.001) changes in cylindrical prescription over the follow-up periods. * Six-month first follow-up, ** 12-month second follow-up.

Mean difference of cylindrical prescription	Mean difference ± Standard deviation		95% confidence interval of the difference		t-value	Sig. (two-tailed)
			Lower	Upper		
Cylinder at presentation vs. cylinder at first follow-up (180 days*)	RE	0.25 ± 0.25	0.22	0.31	12.35	<0.001
	LE	0.24 ± 0.24	0.20	0.28	11.36	
Cylinder at first follow-up vs. cylinder at second follow-up (360 days**)	RE	0.11 ± 0.18	0.08	0.14	7.3	
	LE	0.14 ± 0.21	0.10	0.17	7.58	
Corneal cylinder at presentation vs. first follow-up	RE	0.26 ± 0.24	0.20	0.30	12.86	
	LE	0.26 ± 0.26	0.21	0.30	11.72	
Corneal cylinder at first follow-up vs. second follow-up	RE	0.13 ± 0.19	0.09	0.16	7.7	
	LE	0.13 ± 0.21	0.10	0.17	7.5	

**Table 5:** Odds among the types of astigmatism and amblyopia response to intervention This table shows the odds of amblyopia responding to intervention across different types of astigmatism. The results indicate no statistically significant differences in treatment response among the various astigmatism types.

Type of astigmatism	Achieving amblyopia-free status		Odds	CI	p-value
	No	Yes			
Simple myopic astigmatism	8	13	1.122	0.4128-3.0494	0.8214
Compound myopic astigmatism	8	11	0.9494	0.3402-2.6496	0.921
Simple hypermetropic astigmatism	2	2	0.4603	0.0723-2.9297	0.4113
Compound hypermetropic astigmatism	9	8	0.6138	0.2119-1.7777	0.3683
Mixed astigmatism	29	42	Reference value		

## Discussion

The current study included a group of children of both genders to investigate the visual characteristics and refractive errors in the context of MA. The study explored the findings and offered valuable insights into MA and astigmatism based on foci and loci in children aged 4-11 years and their response to only optical amblyopia therapy. The current study included 57.86% males and 42.14% females. Although there is a difference in the numbers of both genders, the values are not statistically significant and are in line with the published data [12-14]. The potential reason for this difference may be societal discrimination and the provision of equal facilities among male and female participants.

The current study revealed a noticeable improvement in corrected visual acuity following spectacle intervention (Table 1). Previously published studies showed that the mean uncorrected visual acuity before starting the optical corrections ranged from 0.35 to 0.72 Log MAR in ametropic amblyopic cases [15,16]. This study showed that visual acuity improved with a mean value of 0.35 log MAR. The mean difference in corrected and uncorrected visual acuity was 0.38 Log MAR, which showed visual acuity improvement with spectacles and is statistically significant. The current study revealed that there was a mean difference of 1.4 log MAR on the first visit and 1.18 log MAR on the second visit compared with corrected visual acuity at presentation. Hence, an effective optical treatment results in MA. The results of corrected visual acuity are synchronous with previously published studies [7,6,17]. The study showed that the successful amblyopia treatment rate was 57.57% with optical correction over one year. The remaining 43.3% showed improvement in visual acuity with some degree of amblyopia. A probable reason for this is the anisometropic amblyopic condition, where a difference between the two eyes leads to unsuccessful results. This study reflects that MA can be treated if astigmatism is corrected with an exact prescription. The results of the visual outcome depend upon the age of onset and the correction of refractive error. In this study, most of the participants are within the range of 4-7 years of age, which is the critical period for visual development. As they were corrected earlier, the visual improvement was significant. Thus, visual improvement is highly likely if corrections are made early. The results were similar to the previously published studies [17-19].

The current study declared that most cases were under the rule of astigmatism. Further classification showed that mixed astigmatism was more common, followed by myopic astigmatism, with HA being the least common (Figure 2). Harvey showed equivalent results [17]. However, Dobson et al. reported results that contradicted our study results; they showed that HA was more common than myopic and mixed astigmatism. They also found that there was no statistical difference associated with MA in other myopic or mixed astigmatism (M/MA) and HA [18]. The probable cause of discrepancies in these studies may be due to HA that may arise from the differences in how individuals accommodate, leading to varying experiences of blur. Some uncorrected hyperopic astigmats tend to focus on the less hyperopic meridian, while others adjust their focus to fall somewhere in the middle, blurring both stimulus orientations. This phenomenon decreases the tendency to develop meridional blur, thus reducing the tendency to develop MA. On the other hand, it is conceivable that myopic and mixed astigmats undergo different patterns of astigmatic blur formation due to variations in refractive error, leading to MA.

The current study showed that the mean astigmatic refractive error was -3.44 and -3.48 DC, respectively. There was no significant difference in pre- and post-cycloplegic refraction because most astigmatic cases were corneal and had a little internal astigmatism. There are multiple factors associated with the development of the rule of astigmatism. One of the reasons is the lower gauze while using gadgets and the low tensile strength of the cornea. Published data showed that early exposure to electronic gadgets leads to the development of astigmatic refractive error [20]. This study revealed a significant difference in the magnitude of astigmatism over one year. The mean difference in astigmatism is 0.24 DC in the six-month follow-up and 0.36 DC after one year (Table 4). This astigmatism was corneal. Studies showed that the cornea had low tensile strength in the early developmental phase of life. The lid thickness and powerful orbicularis oculi were the reasons for changing corneal shape, leading to steeper vertical curvature [21].

The study's findings suggest a promising avenue for the effective management of MA with optical correction of astigmatism. The study highlighted the substantial impact of early detection and intervention on visual acuity outcomes in the case of MA. The predominance of with-the-rule astigmatism among participants, coupled with the observed significant improvement in visual acuity following spectacle intervention, underscores the critical role of precise optical correction in the treatment of amblyopia. Additionally, the variation in astigmatism types and their association with MA highlight the complexity of astigmatic refractive errors and the necessity for tailored treatment approaches. It is early to say that exposure to electronic gadgets and the development of astigmatic refractive errors are correlated; however, the need for further research into preventative measures can mitigate the risk factors associated with early childhood astigmatism. The study also shows that factors such as low corneal tensile strength and eyelid pressure may influence the progression of astigmatism. It is suggested that a multidisciplinary approach, incorporating both optical correction and potentially behavioral interventions, could enhance treatment efficacy.

Limitations of the study

The limitations of the study include the lack of adaptation time for assessing visual acuity after intervention, a limited sample size, and the absence of comparisons among other amblyopic management techniques.

## Conclusions

The study concluded that early and precise optical intervention is very important for treating MA in children aged 4 to 11, especially those with astigmatic refractive errors. Significant improvement in visual acuity was observed after one year of spectacle use. The study highlights a predominance of with-the-rule and mixed astigmatism but notes that the astigmatism type did not significantly impact visual outcomes. Early intervention, especially within the critical period of visual development, was associated with notable improvement in visual acuity. The progressive increase in cylindrical prescriptions over time indicates the dynamic nature of astigmatic errors. The study also suggests that factors such as corneal tensile strength and electronic device use may influence astigmatism, warranting further investigation. Overall, the results support timely, accurate optical correction and ongoing monitoring as ways to better treat amblyopia. A multidisciplinary approach might also provide extra benefits.
